# The Association Between Vitamin D Deficiency and Diabetes in Adult African Americans and Whites: An NHANES Study

**DOI:** 10.1007/s40615-024-02144-4

**Published:** 2024-09-23

**Authors:** Alula Hadgu, Fengxia Yan, Robert Mayberry

**Affiliations:** Department of Community Health and Preventive Medicine, Morehouse School of Medicine, 720 Westview Dr., SW, Atlanta, GA 30310 USA

**Keywords:** Vitamin D deficiency, Diabetes, NHANES, Propensity score analysis, Health disparity

## Abstract

**Objective:**

The primary objective of this cross-sectional study is to investigate the association between vitamin D deficiency (VDD) and diabetes and see if this association is the same for adult (age ≥ 20) African Americans (AAs) and Whites. The secondary objective is to examine the distribution of the 25-hydroxyvitamin D test among AAs and Whites and to evaluate the appropriateness of using the same cut-off point for both groups to diagnose VDD.

**Methods:**

Our analysis is based on the 2011–2014 National Health and Nutrition Examination Surveys (NHANES). We used two common propensity score adjustment methods to analyze the data—propensity score matching (PSM) and the inverse probability of treatment weighting (IPTW).

**Results:**

The prevalence of diabetes for AAs and Whites was 12.27% (95% CI, 10.47–14.07%) and 7.24% (95% CI, 6.35–8.13%), respectively. The prevalence of VDD for AAs and Whites was 65.29% (95% CI, 62.01–68.58%) and 19.49% (95% CI, 16.53–22.45%), respectively. Under PSM, the odds ratios for the diabetes-VDD association for AAs and Whites were 0.94 (95% CI, 0.70–1.27) and 2.16 (95% CI, 1.49–3.13), respectively. Under IPTW, the VDD-diabetes odds ratios for AAs and Whites were 0.83 (95% CI, 0.64–1.10) and 2.35 (95% CI, 1.67–3.30), respectively. Our results further demonstrate that the 25-hydroxyvitamin D measurements are significantly different for AAs and Whites across the general population, as well as the vitamin D–sufficient and vitamin D–deficient populations.

**Conclusion:**

The prevalence of VDD and diabetes was higher for AAs compared to Whites. However, VDD was associated with increased diabetes risk for Whites but not for AAs. Though more research is needed to explain why this is the case, a reason for this may be that the 25-hydroxyvitamin D test or its associated cut-off point for defining VDD may not accurately reflect the vitamin D status among AAs.

**Supplementary Information:**

The online version contains supplementary material available at 10.1007/s40615-024-02144-4.

## Background

Diabetes mellitus (diabetes) is a common disease in the United States (US). The crude overall prevalence of diagnosed diabetes in adults ≥20 years is 9.6% [1]. An additional estimated 2.9% of the US population has undiagnosed diabetes, amounting to a total prevalence of 12.5% or 28.2 million in the US civilian noninstitutionalized population [1]. Diabetes is the 7^th^ leading cause of death in the US [2]. The prevalence of diagnosed diabetes increased by 42% from 2001 to 2020 [3]. According to the Centers for Disease Control and Prevention (CDC) researchers, unless prevention measures are taken now, as many as one in three adult Americans will develop diabetes by 2050 [4].

Diabetes is an epidemic in African Americans (AAs). AAs are 75% more likely to have been diagnosed with diabetes compared to Whites [3]. Complications of diabetes include cardiovascular diseases, neuropathy, nephropathy, retinopathy, foot diseases, and other conditions. AA adults with diabetes have higher rates of albuminuria, retinopathy, and worse glycemic control compared to Whites [5].

The prevalence of VDD is also much higher in AAs than in Whites. The prevalence of low serum 25-hydroxyvitamin D concentration (<30 nmol/L) in AAs is 37.4% with a 95% CI (32.2–42.9) [6]. The corresponding figure for Whites is 4.0% with a 95% CI (3.1–5.1). Similarly, the mean level of serum 25-hydroxyvitamin D concentration for AAs is 47.0 (95% CI, 42.6–51.3) [6]. The corresponding figure for Whites is 75.3 (95% CI, 72.9–77.7), indicating a marked difference. Note that when VDD is defined as a serum 25-hydroxyvitamin D concentration ≤ 20 ng/mL (50 nmol/L), the prevalence of VDD in AAs is 82% [7]. What is interesting and paradoxical is that despite markedly low levels of vitamin D in AAs, the incidence of conditions associated with VDD, such as falls, fractures, or osteopenia, is significantly lower in the AA population compared to their White American counterparts with similar vitamin D levels [8]. Brown et al. [8] stated that even though variables such as adiposity, skin pigmentation, vitamin D–binding protein polymorphisms, and genetics can contribute to differences in vitamin D levels in AAs vs. White Americans, no one factor alone could fully explain the vitamin D paradox in AAs, indicating that this is a true paradox. The reason why AAs have a higher proportion of VDD is partly attributed to melanin which can act as a natural sunscreen and limit vitamin D production in people with dark skin [9]. Other reasons why AAs have high levels of VDD include lactose intolerance [10] and obesity [11].

The association between VDD and diabetes is based on the postulation that vitamin D can improve the body’s sensitivity to insulin, the hormone responsible for regulating blood sugar levels, and thus reduce the risk of diabetes. Berridge [12] described that diabetes begins with the onset of insulin resistance. To counteract this, β-cells release more insulin, preventing hyperglycemia. However, prolonged hyperactivity leads to excessive Ca^2+^ and reactive oxygen species (ROS) signaling, resulting in β-cell death and the progression of diabetes. Vitamin D deficiency (VDD) contributes to both initial insulin resistance and subsequent β-cell death. Vitamin D reduces inflammation, a key factor in insulin resistance, and helps maintain normal resting levels of Ca^2+^ and ROS, which are elevated in β-cells during diabetes [12].

Observational studies [13–17] and meta-analyses [18, 19] have shown that VDD increases the risk of developing diabetes. An observational study from the Nurses’ Health Study [13] showed that VDD was associated with diabetes. Using the National Health and Nutrition Examination Survey III conducted between 1988 and 1994, Scragg et al. [14] demonstrated that there is a strong inverse relationship between low levels of 25(OH)D and diabetes prevalence for Whites and Hispanics, but not in AAs. Kayaniyil et al. [15] showed that vitamin D was significantly associated with insulin resistance and β-cell function. They concluded that vitamin D levels may play a significant role in diabetes. A study by Park et al. [16] suggested that people deficient in vitamin D may be at much greater risk of developing diabetes. These authors stated that there was an inverse dose-response gradient between 25(OH)D concentration and risk of diabetes with a *p*-value for trend of 0.005. Another observational study showed that increasing vitamin D serum to normal levels can result in a 55% relative reduction in the risk of developing diabetes [17]. Ashraf et al. showed that 78% of obese AA female adolescents were vitamin D deficient, and participants with low vitamin D levels had greater insulin resistance [17].

The relationship between VDD and diabetes is not, however, unequivocal. Results from randomized clinical trials of vitamin D and diabetes to date are inconclusive and contradictory. Many of these studies were conducted based on small sample sizes or inadequate amounts of vitamin D. In a meta-analysis of 35 randomized clinical trials (*n*=43,407 patients), Seida et al. [18] concluded that there was no statistically significant effect of vitamin D3 supplementation on glucose homeostasis or diabetes prevention. On the contrary, Pitas [19] et al., in another meta-analysis, showed that vitamin D and calcium insufficiency may negatively influence glycemia, whereas vitamin D and/or calcium supplementation may be beneficial in optimizing glucose metabolism. In a RCT of 96 patients, Lemieux et al. [20] demonstrated that in individuals at high risk of diabetes or those newly diagnosed with type 2 diabetes, taking vitamin D supplements for 6 months significantly improved peripheral insulin sensitivity and β-cell function. However, in a large (*n*=2423) and diverse randomized clinical trial, Pitas et al. [21] showed that taking a daily vitamin D supplement does not prevent type 2 diabetes in high-risk adults.

This cross-sectional study’s primary objective is to fill a critical void in our understanding of the association between VDD and the risk of diabetes among a representative sample of AAs and Whites. Thus, given the fact that the association between vitamin D and diabetes is equivocal and that AAs are a high-risk group for the development of diabetes and have a high rate of VDD, it is important to conduct additional studies by applying statistical models useful in reducing selection bias and improve causal inference in a representative national complex sample survey of the US population with a large sample size of AAs and Whites to make comparisons. Thus, in this study, we wish to answer the following questions: Is VDD associated with diabetes in adults (age ≥ 20)? And if so, is the association the same for AAs and Whites? Our secondary objective is to assess the distribution of the 25-hydroxyvitamin D test in AAs and Whites and determine whether it’s appropriate to apply the same cut-off point for both AAs and Whites when diagnosing individuals as vitamin D deficient.

## Methods

### Survey Design

In this work, we used the 2011–2014 NHANES to study the association between VDD and diabetes. Though the survey was conducted between 2011 and 2014, vitamin D measurements were not included until 2018 [22]. The NHANES is a cross-sectional complex sample survey designed to provide national statistical estimates on the health and nutrition of the US civilian noninstitutionalized population. The sampling design is a stratified, multistage, probability cluster design. The survey was conducted by the CDC’s National Center for Health Statistics.

### Statistical Analysis

We performed statistical analyses using SAS software for complex sample surveys (SAS Institute, Cary, NC). Analyses performed with SAS accounted for the complex survey design by incorporating the survey weights. Thus, data were weighted to account for the unequal probability of selection and nonresponse during the interview and examination process. Baseline characteristics of the unmatched and matched samples with VDD and vitamin D–sufficient groups are compared using weighted chi-squared tests and ANOVA tests as appropriate. Descriptive statistics using percentages or means with standard deviations (SD) are provided.

In this study, we used propensity score methods [23, 24] to analyze the data. In observational studies, like the NHANES, there are often significant differences in covariate distribution between the VDD and vitamin D–sufficient groups. Thus, such differences must be adjusted using models that can improve causal inference, like propensity score analysis, to reduce selection bias inherent in NHANES-type studies and estimate the effect of VDD on diabetes risk.

Here, the propensity score is the probability that an individual is VDD given his/her covariate information. The variables for our propensity score models were selected a priori based on the published literature and/or on subject matter knowledge [25]. Such variables or covariates include demographic, clinical, and behavioral variables associated with both the treatment (VDD) and the outcome (diabetes) (Appendix). Our outcome variable, the presence or absence of diabetes, was not included in developing our propensity score models.

We used two common propensity adjustment methods to estimate the effect of VDD on diabetes. We first used propensity score matching (PSM) using the greedy matching algorithm [26], where we matched one VDD individual for each vitamin D–sufficient individual to create matched samples. Then, we used the inverse probability of treatment weighting (IPTW) method to obtain the estimated effect of VDD on our outcome variable, the presence of diabetes. These two propensity score adjustment methods are the two most popular methods used in propensity score analysis and are useful to address confounding by indication in observational studies [27]. We used both the PSM and IPTW algorithms because we wanted to cross-validate the findings and ensure robustness. Note that the PSM method estimates the average VDD effect for those individuals with VDD whereas the IPTW method estimates the average VDD effect for the entire study population. As a result, in the PSM approach, unmatched individuals are excluded from the analysis, reducing the sample size markedly, whereas in the IPTW approach, all eligible participants are included in the analysis.

We performed covariate balance using standardized mean differences (SMD) for all the variables used to develop our propensity score models. Categorical or ordinal variables were transformed into indicator variables to enable the assessment of covariate balance using SMD. We used logistic regression to estimate the effect of VDD on diabetes. All parameter estimates are based on complete case data analysis.

We defined someone as having diabetes if his or her glycohemoglobin level (A1C) is equal to or greater than 6.5%. Consistent with the clinical practice guidelines of the Endocrine Society Taskforce on vitamin D [28], we defined someone as VDD if his or her serum 25-hydroxyvitamin D level was less than 50 nmol/L.

## Results

Supplemental Figure 1 shows the flowchart for the selection of study participants. A total of 6530 (2334 VDD and 4196 vitamin D sufficient) individuals in this representative national sample were eligible for this study. In this sample, 4194 participants were White, and 2336 were AAs. Table 1 shows the distribution of demographic, clinical, and behavioral variables for the VDD and vitamin D–sufficient groups before matching. Of the 31 variables in Table 1, 16 were statistically significantly different (*p* < 0.05). VDD individuals were younger, had a higher proportion of AAs, and had a higher prevalence of clinical and behavioral conditions, including high blood pressure, poor general health conditions, congestive heart failure, having little interest in doing things, feeling down, depressed, or helpless.

**Table 1:** Baseline covariate distribution by vitamin d status—unmatched data

Variable #	Variable name	Vitamin D deficient *n* = 2334	Vitamin D sufficient *n* = 4196	*p*-value
	Demographic			
1	**Gender**			0.3623
	Male	46.6%	48.3%	
	Female	53.4%	51.7%	
2	**Age in Years**			0.0002
	Mean (SE)	46.5 (0.69)	49.8 (0.53)	
3	**Race**			< 0.0001
	White	64.5	93.4	
	AA	35.5	6.6	
4	**Marital status**			< 0.0001
	Married	42.2%	59.5	
	Widowed	6.7	6.1	
	Divorced	14.5	10.7	
	Separated	3.2	1.3	
	Never married	26.4	15.9	
	Living with partner	7.1	6.5	
5	**BMI**			< 0.0001
	Mean (SE)	31.2 (0.20)	28.3 (0.15)	
6	**Citizenship status**			0.3916
	Citizen	97.8%	98.3	
	Not-citizen	2.2	1.7	
7	**Education level**			< 0.0001
	< 9th grade	2.6%	2.2	
	9–11 grade	12.5	8.2	
	High school	24.5	20.3	
	Some college	37.5	32.5	
	College graduate	23.0	36.9	
8	**Annual family income**			< 0.0001
	$0 to $4999	4.2%	2.2	
	$5000 to $9999	5.7	2.9	
	$10,000 to $14,999	6.8	5.3	
	$15,000 to $19,999	6.8	4.1	
	$20,000 to $24,999	7.7	5.5	
	$25,000 to $34,999	12.4	8.9	
	$35,000 to $44,999	10.0	9.0	
	$45,000 to $54,999	7.5	7.9	
	$55,000 to $64,999	6.5	6.1	
	$65,000 to $74,999	5.3	5.8	
	$20,000 and over	2.4	2.2	
	Under $20,000	1.0	0.5	
	$75,000 to $99,999	8.4	11.1	
	$100,000 and over	15.4	28.5	
	Medical			
9	**Ever told you had high blood pressure**			0.0488
	Yes	39.4%	34.6	
	No	60.6	65.4	
10	**Doctor told you—high cholesterol level**			0.0081
	Yes	32.5%	37.9	
	No	67.5	62.1	
11	**Diastolic blood pressure**			< 0.0053
	Mean (SE)	71.3 (0.56)	70.1 (0.42)	
12	**Systolic blood pressure**			< 0.0001
	Mean (SE)	124.2 (0.66)	121.1(0.41)	
13	**General health condition**			< 0.0001
	Excellent	6.8%	12.6	
	Very good	25.1	37.6	
	Good	44.9	37.2	
	Fair	18.9	11.2	
	Poor	4.2	1.5	
14	**Total cholesterol**			0.0364
	Mean (SE)	190.5 (1.3)	193.4 (0.86)	
15	**Ever told you had cancer/malignancy**			0.0059
	Yes	9.7%	12.7	
	No	90.3	87.3	
16	**Ever told had congestive heart failure**			0.0394
	Yes	4.1%	2.6	
	No	95.9	97.4	
17	**Ever told you had coronary heart dx**			0.7712
	Yes	3.9%	3.8	
	No	96.1	96.2	
18	**Ever told you had angina**			0.1611
	Yes	2.9%	2.3	
	No	97.1	97.7	
19	**Ever told you had heart attack**			0.0685
	Yes	4.4%	3.4	
	No	95.6	96.6	
20	**Ever told you had a stroke**			0.1258
	Yes	3.7%	2.9	
	No	96.3	97.1	
21	**Ever told you had emphysema**			0.2656
	Yes	2.8%	2.0	
	No	97.2	98.0	
	Behavioral			
22	**Have little interest in doing things**			0.0376
	Not at all	73.9%	79.3	
	Several days	16.9	13.8	
	> half the days	5.2	4.0	
	Nearly every day	4.0	3.0	
23	**Feeling down, depressed, or helpless**			0.0048
	Not at all	73.7%	79.5	
	Several days	17.9	15.1	
	> half the days	4.9	2.9	
	Nearly every day	3.5	2.5	
24	**Trouble sleeping or sleeping too much**			0.0200
	Not at all	60.5%	63.1	
	Several days	21.3	22.8	
	> half the days	7.7	6.7	
	Nearly every day	10.4	7.4	
25	**Feeling bad about yourself**			0.0760
	Not at all	81.7%	84.2	
	Several days	11.3	11.0	
	> half the days	4.2	2.5	
	Nearly every day	2.8	2.3	
26	**Trouble concentrating on things**			0.5032
	Not at all	82.5%	84.1	
	Several days	10.6	10.3	
	> half the days	3.4	3.1	
	Nearly every day	3.4	2.6	
27	**Thought you would be better off dead**			0.4099
	Not at all	96.4%	97.0	
	Several days	2.1	2.3	
	> half the days	0.7	0.3	
	Nearly every day	0.9	0.4	
28	**Vigorous work activity**			0.1755
	Yes	17.6%	19.3	
	No	82.4	80.7	
29	**Moderate work activity**			0.2780
	Yes	35.0%	37.4	
	No	65.0	62.6	
30	**Walk or bicycle**			0.0741
	Yes	27.7%	24.8	
	No	72.3	75.2	
31	**Smoked at least 100 cigarettes in life**			0.6246
	Yes	46.8%	46.0	
	No	53.2	54.0	

Table 2 shows the standardized mean differences for the matched and unmatched samples. After matching, all the variables attained covariate balance (the absolute standardized mean differences after matching were less than 0.10). Similarly, for the IPTW model, all the absolute standardized mean differences were less than 0.10, indicating good covariate balance. Supplemental Figures 2 and 3 show the plots of the standardized mean differences for the PSM and IPTW models, respectively, indicating covariate balance.

**Table 2:** Standardized mean differences (SMD) for the matched and unmatched samples

Variable #	Variable name	VDD Vitamin D deficient n = 1306	Vit. D sufficient n = 1306	SMD-matched Standardized mean difference (matched)	SMD-unmatched Standardized mean difference (unmatched)
	**Demographic**				
1	**Gender**				
	Male	48.01	50.21		
	Female	52.00	49.79	0.0000	− 0.01439
2	**Age in years**				
	Mean (SE)	47.99 (0.81)	45.70 (9.89)	− 0.01060	− 0.27665
3	**Race**				
	White	77.75	79.23		
	AA	22.25	20.75	0.01373	− 0.92918
4	**Marital status**				
	Married	47.42	48.06		
	Widowed	6.70	5.86	0.00000	0.02542
	Divorced	14.87	14.37	− 0.00908	− 0.07623
	Separated	1.98	1.66	0.00000	− 0.15041
	Never married	22.17	23.89	0.02918	− 0.28928
	Living with partner	6.87	6.16	− 0.0300	− 0.02064
5	**BMI**				
	Mean (SE)	30.54 (0.29)	29.52 (0.31)	0.06107	0.31510
6	**Citizenship status**				
	Citizen	97.94	97.89		
	Not citizen	2.06	2.11	0.00000	− 0.05294
7	**Education level**				
	< 9th grade	2.0	1.94		
	9th to 11th grade	10.67	10.80	− 0.01371	− 0.11827
	HS graduate	24.50	22.59	− 0.01610	− 0.11825
	Some college	36.36	39.20	0.00802	− 0.09324
	College graduate	26.51	25.47	0.01624	0.31129
8	**Annual family income**				
	$0 to $4999	3.72	3.31		
	$5000 to $9999	4.35	4.14	− 0.01983	− 0.11205
	$10,000 to $14,999	5.74	7.36	0.02423	− 0.05221
	$15,000 to $19,999	5.04	5.83	0.03819	− 0.09773
	$20,000 to $24,999	7.18	6.53	− 0.03555	− 0.06647
	$25,000 to $34,999	12.84	11.28	− 0.00471	− 0.06449
	$35,000 to $44,999	10.23	9.90	− 0.00259	− 0.03880
	$45,000 to $54,999	7.57	8.68	0.00610	0.01144
	$55,000 to $64,999	7.44	7.28	0.01028	0.02855
	$65,000 to $74,999	4.64	6.56	0.01826	0.03083
	$20,000 and over	2.30	1.54	− 0.01985	− 0.01540
	Under $20,000	0.63	0.64	0.03431	− 0.01945
	$75,000 to $99,999	9.86	9.55	0.00839	0.10180
	$100,000 and over	18.45	17.40	− 0.0232	0.25297
	**Clinical**				
9	**Ever told you had high blood pressure**				
	Yes	39.47	35.22		
	No	60.53	64.78	0.00156	0.04735
10	**Doctor told you—high cholesterol level**				
	Yes	35.32	29.00		
	No	64.68	71.05	0.03395	− 0.18223
11	**Diastolic: blood pressure**				
	Mean (SE)	71.36 (0.67)	71.00 (0.55)	− 0.02165	0.07522
12	**Systolic: blood pressure**				
	Mean (SE)	123.91 (0.89)	121.94 (0.50)	− 0.00171	0.11941
13	**General health condition**				
	Excellent	7.78	9.08		
	Very Good	28.83	29.25	− 0.01856	0.21301
	Good	45.47	47.22	0.02793	− 0.12871
	Fair	17.91	17.45	− 0.02572	− 0.18126
	Poor				
14	**Total cholesterol**				
	Mean (SE)	192.20 (1.82)	191.57 (1.81)	0.00172	− 0.08457
15	**Ever told you had cancer or malignancy**				
	Yes	11.10	10.03		
	No	88.90	90.00	− 0.01778	0.18248
16	**Ever told had congestive heart failure**				
	Yes	3.31	2.30		
	No	96.69	97.70	0.00428	0.01027
17	**Ever told you had coronary heart disease**				
	Yes	4.00	3.32		
	No	96.02	96.68	0.01568	− 0.03646
18	**Ever told you had angina/angina pectoris**				
	Yes	2.61	2.20		
	No	97.39	97.80	− 0.00500	− 0.01576
19	**Ever told you had heart attack**				
	Yes	3.95	2.86		
	No	96.05	97.14	0.01926	0.01084
20	**Ever told you had a stroke**				
	Yes	3.16	2.95		
	No	96.84	97.05	− 0.01128	− 0.00622
21	**Ever told you had emphysema**				
	Yes	2.74	2.00		
	No	97.26	98.00	0.01624	0.02707
	**Behavioral**				
22	**Have little interest in doing things**				
	Not at all	77.09	77.70		
	Several days	15.21	15.00	− 0.01477	− 0.09136
	> half the days	4.52	4.37	− 0.01052	− 0.03968
	Nearly every day	3.18	2.93	− 0.00852	− 0.02428
23	**Feeling down, depressed, or helpless**				
	Not at all	76.32	77.22		
	Several days	17.40	17.16	− 0.02690	− 0.07297
	> half the days	3.75	2.75	− 0.02389	− 0.05129
	Nearly every day	2.52	2.86	− 0.01818	− 0.06854
24	**Trouble sleeping or sleeping too much**				
	Not at all	61.33	60.05		
	Several days	21.40	22.41	0.03392	0.04388
	> half the days	8.03	7.13	− 0.00596	0.01597
	Nearly every day	9.23	10.41	0.00816	− 0.06300
25	**Feeling bad about yourself**				
	Not at all	83.37	85.21		
	Several days	10.78	9.60	− 0.03935	0.01227
	> half the days	3.40	3.22	− 0.01277	− 0.06035
	Nearly every day	2.45	2.00	− 0.02952	− 0.01700
26	**Trouble concentrating on things**				
	Not at all	84.62	84.72		
	Several days	9.41	9.68	− 0.00508	− 0.00591
	> half the days	3.13	3.00	− 0.00508	0.02489
	Nearly every day	2.84	2.61	− 0.00452	− 0.03143
27	**Thought you would be better off dead**				
	Not at all	96.90	96.94		
	Several days	1.70	1.96	− 0.01637	0.04052
	> half the days	0.70	0.47	− 0.01109	− 0.02247
	Nearly every day	0.70	0.63	0.00000	− 0.04688
28	**Vigorous work activity**				
	Yes	19.68	20.78		
	No	80.32	79.22	− 0.01377	− 0.06004
29	**Moderate work activity**				
	Yes	36.70	38.98		
	No	63.30	61.01	− 0.01277	− 0.07015
30	**Walk or bicycle**				
	Yes	27.46	26.27		
	No	72.54	73.73	− 0.00344	0.07889
31	**Smoked at least 100 cigarettes in life**				
	Yes	48.41	49.93		
	No	51.59	50.07	0.01839	0.01430

### Prevalence of Diabetes and VDD

The prevalence of diabetes for AAs and Whites was 12.27% (95% CI, 10.47–14.07%) and 7.24% (95% CI, 6.35–8.13%), respectively. Similarly, the prevalence of VDD for AAs and Whites were 65.29% (95% CI, 62.01–68.58%) and 19.49% (95% CI, 16.53–22.45%), respectively.

### Estimated VDD Effect on Diabetes Under the PSM and IPTW Models

Table 3 shows regression parameter estimates and their associated standard errors for VDD, race, and the interaction of VDD and race for both the PSM and IPTW algorithms. Note that the interaction term is statistically significant indicating that the effect of VDD on diabetes is not the same for AAs and Whites. Table 4 shows the odds ratio estimates for the effect of VDD on diabetes for AAs and Whites. Under the PSM algorithm with greedy matching, the VDD-diabetes odds ratios for AAs and Whites were 0.94 (95% CI, 0.70–1.27) and 2.16 (95% CI, 1.49–3.13), respectively (Table 4).

**Table 3:** Regression parameter estimates and their standard errors under the PSM and IPTW algorithms

Algorithm	Parameter	Estimate	SE	*p*-value
PSM	Intercept	− 2.72	0.17	< 0.0001
	VDD	0.77	0.18	0.0002
	Race	0.79	0.22	0.0009
	VDD*Race	− 0.83	0.21	0.0004
IPTW	Intercept	− 2.79	0.08	< 0.0001
	VDD	0.85	0.17	< 0.0001
	Race	0.87	0.14	< 0.0001
	VDD*Race	− 1.03	0.18	< 0.0001

**Table 4:** Estimated vitamin D deficiency effect on diabetes for African Americans and Whites

Model	Variable	Odds ratio	95% CI	*n*
Crude (unadjusted)	African American	0.81	0.62–1.06	6519
	White	2.13	1.62–2.80	
PS matched	African American	0.94	0.70–1.27	2612
	White	2.16	1.49–3.13	
IPTW	African American	0.83	0.64–1.10	5275
	White	2.35	1.67–3.30	

Similarly, under the IPTW model, the VDD-diabetes odds ratios for AAs and Whites were 0.83 (95% CI, 0.64–1.10) and 2.35 (95% CI, 1.67–3.30), respectively.

### Post HocResults

As part of post hoc analyses, we investigated the association between severe VDD (SVDD) and diabetes. Researchers [4, 28–31] have defined someone to have SVDD if his or her serum 25-hydroxyvitamin D level is less than 30 nmol/L. If we use the 30 nmol/L cut-off to define SVDD, then the prevalence of SVDD in AAs and Whites are 28.85% (95% CI, 24.75–32.96%) and 4.36% (95% CI, 3.30–5.41%), respectively. Table 5 shows the odds ratio estimates for the effect of SVDD on diabetes for AAs and Whites. Under the PSM algorithm, the SVDD-diabetes odds ratios for AAs and Whites were 1.04 (95% CI, 0.66–1.65) and 2.57 (95% CI, 1.66–6.26), respectively. Similarly, under the IPTW model, the SVDD-diabetes odds ratios for AAs and Whites were 0.99 (95% CI, 0.64–1.53) and 2.29 (95% CI, 1.30–4.03), respectively, which are not markedly different from the VDD-diabetes odds ratios we observed above.

**Table 5:** Estimated severe vitamin D deficiency effect on diabetes for African Americans and Whites

Model	Variable	Odds ratio	95% CI	*n*
Crude (unadjusted)	African American	0.91	0.62–1.35	6519
	White	2.05	1.24–3.39	
PS matched	African American	1.04	0.66–1.65	1254
	White	2.57	1.66–6.26	
IPTW	African American	0.99	0.64–1.53	5275
	White	2.29	1.30–4.03	

### Distribution of 25-Hydroxyvitamin D Test Levels for AAs and Whites

**Fig. 1:**
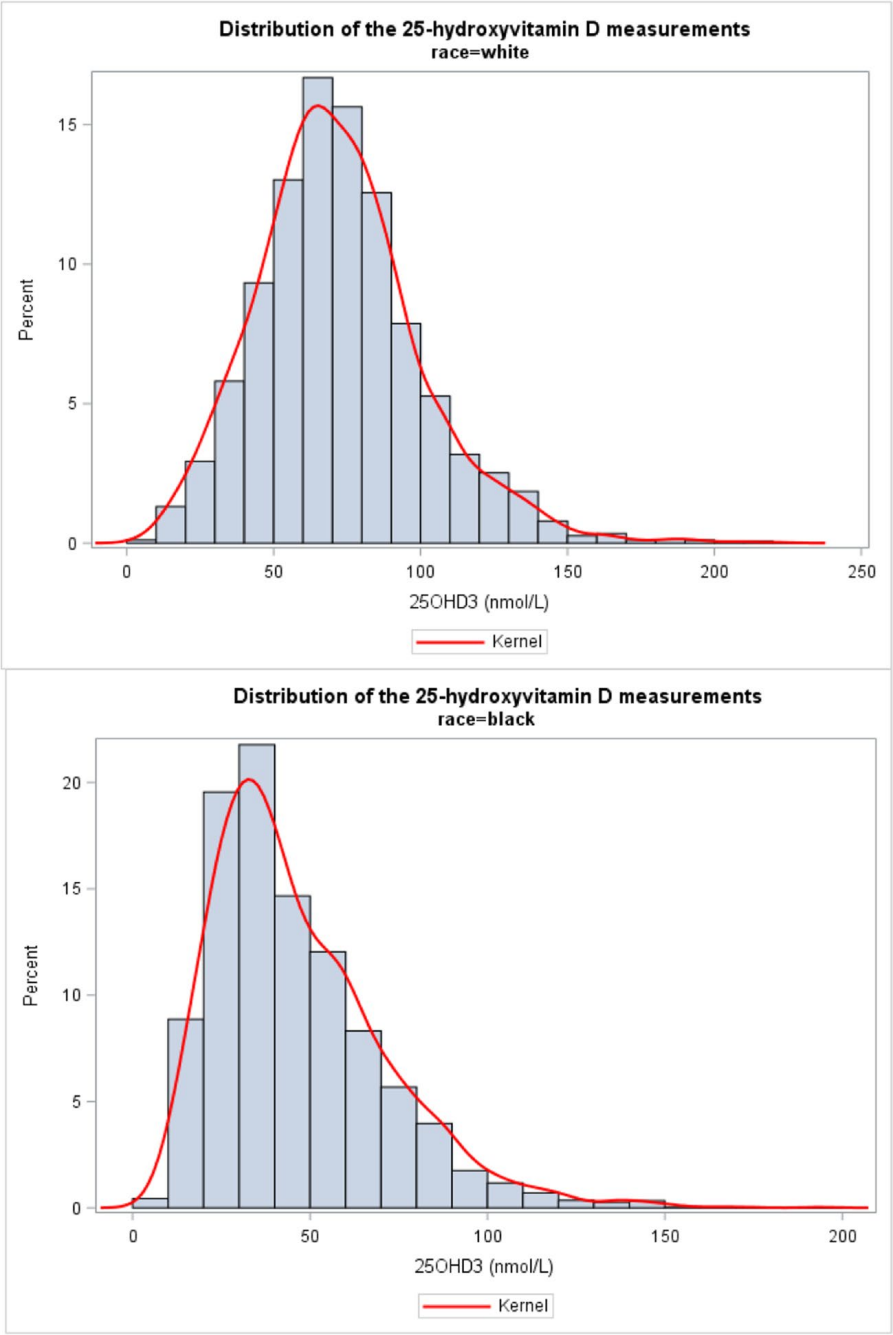
Distribution of the 25-hydroxyvitamin D measurements (LBXVDM3) for Whites (top) and African Americans (bottom)

**Fig. 2:**
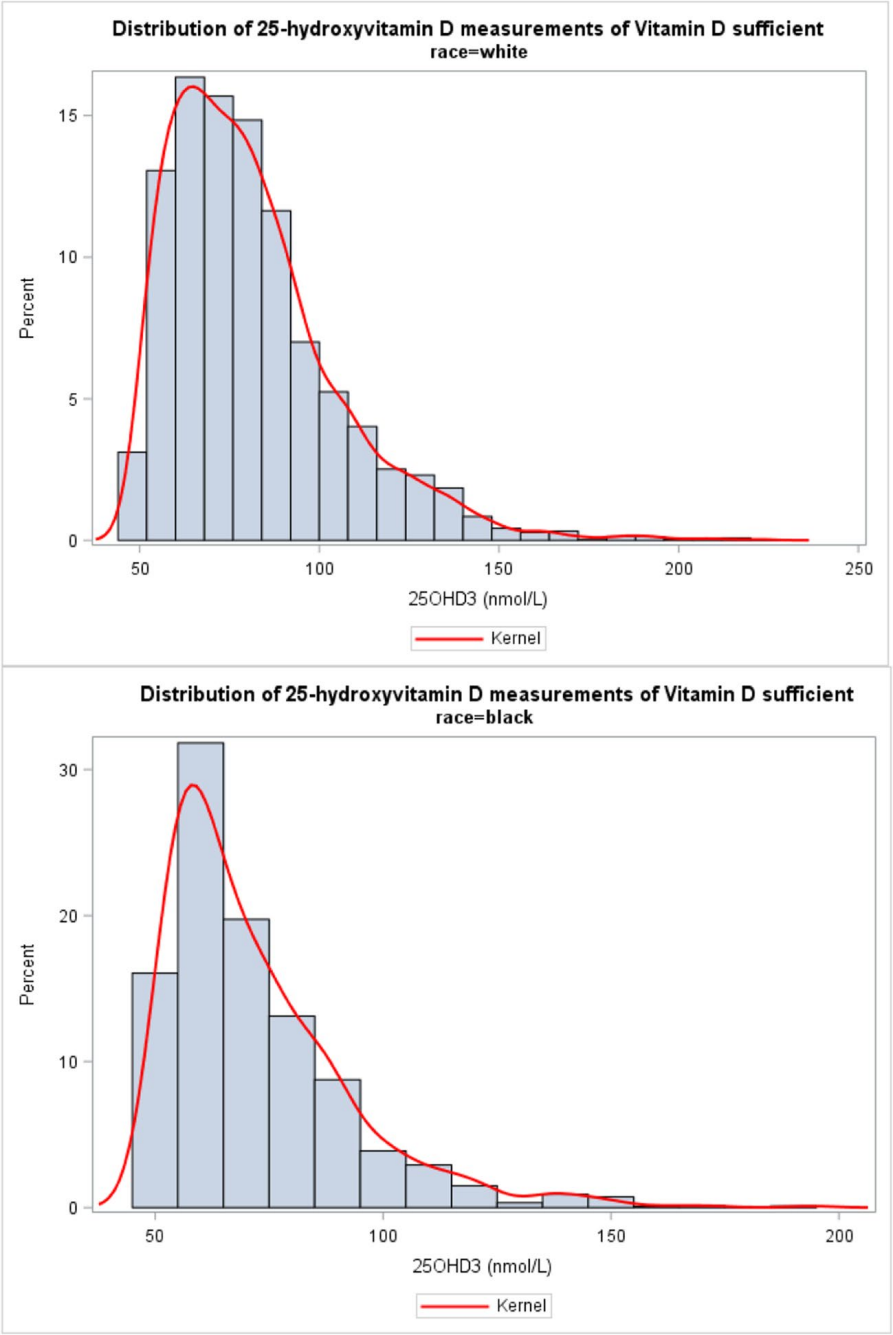
Distribution of 25-hydroxyvitamin D measurements (LBXVDM3) for Whites (top) and African Americans (bottom) for vitamin D–sufficient individuals

Figures 1 and 2 show the 25-hydroxyvitamin D histograms for all individuals and vitamin D–sufficient individuals, respectively. In each population, the distribution of the 25-hydroxyvitamin D measurements for AAs and Whites were significantly different (*p* < 0.05). As shown in Fig. 1 (all individuals), AAs had a markedly and significantly lower 25-hydroxyvitamin D concentration compared to Whites (weighted mean 45.3; 95% CI (43.5–47.2) compared to 73.0; 95% CI, 71.1–75.0). Among vitamin D–sufficient individuals (Fig. 2), AAs had a lower 25-hydroxyvitamin D concentration level compared to Whites (weighted mean 71.4, 95% CI 69.9–72.9 compared to 81.7, 95% CI 80.6–82.8). Similarly, among individuals with VDD, AAs had lower 25-hydroxyvitamin D concentration levels (weighted mean 31.5, 95% CI 30.4–32.5 compared to 37.4, 95% CI 36.5–38.1).

## Discussion

The primary objective of this study was to investigate the association between VDD and diabetes in AAs and Whites. Our analysis, using a large representative sample size of the US noninstitutionalized civilian population, demonstrated that even though the prevalence of both VDD and diabetes is higher in AAs than in Whites, VDD was strongly associated with increased diabetes risk for Whites but not for AAs. Our findings in AAs, under the PSM and IPTW algorithms, demonstrate that the VDD-diabetes odds ratios were 0.94 (95% CI, 0.70–1.27) and 0.83 (95% CI, 0.64–1.10), respectively. On the other hand, for Whites, under the PSM and IPTW algorithms, the VDD-diabetes odds ratios were 2.16 (95% CI, 1.49–3.13) and 2.35 (95% CI, 1.67–3.30), respectively. Similarly, SVDD was strongly associated with higher diabetes risk for Whites but not for AAs.

Our results are consistent with the work of Scragg et al. [14]. However, these authors only adjusted for five covariates using multiple linear regression; in contrast, our analysis incorporated a comprehensive adjustment for 31 demographic, clinical, and behavioral variables using propensity score analysis, which can result in less biased and more accurate treatment effect. A recent study [31] on aging, based on a nationally representative sample of 9100 participants in Ireland also demonstrated that VDD was associated with an increased risk of developing diabetes (relative risk ratio of 1.5, 95% CI 1.03–2.18; *p*=0.037). In the prospective analysis part of the study by McCarthy et al., to investigate the association of VDD and diabetes status 4 years later, the authors showed that there was a 62% increased risk (relative risk ratio, 1.62, 95% CI 1.12–2.35; *p*=0.011) for those study participants with vitamin D < 30 nmol/L compared with ≥ 75 nmol/L.

Though our study and others have shown a statistically significant association between VDD and diabetes, there are not many studies that have shown that vitamin D supplementation reduces the risk of diabetes. On the contrary, recent randomized clinical trials demonstrated that vitamin D supplementation did not result in lowering the risk of diabetes [21, 32, 33]. Pittas et al. [21] showed that in individuals at high risk for type 2 diabetes (i.e., persons with prediabetes), vitamin D supplementation at a dose of 4000 IU per day did not result in a statistically significant risk reduction in diabetes compared to placebo. The hazard ratio for vitamin D supplementation versus placebo was 0.88 (95% CI, 0.75–1.04; *p*-value=0.12) [21]. Similarly, another RCT conducted in Norway among primarily White individuals showed that vitamin D supplementation was unlikely to prevent progression from prediabetes to diabetes (hazard ratio 0.90, 95% CI 0.69–1.18, *p*=0.45) [32]. Furthermore, a group of researchers in Japan randomized 1256 adults with prediabetes to vitamin D analog (eldecalcitol) or placebo, and once again vitamin D supplementation did not reduce the risk of diabetes (hazard ratio 0.87; 95% CI, 0.68–1.09) [33]. Note that these studies were based on individuals with a high risk of developing diabetes since all the study participants had prediabetes, whereas our study population is the general noninstitutionalized US population. Secondly, the participants in these RCTs were not selected based on VDD status, whereas in our study the focus was VDD status and diabetes.

The diabetes prevalence in AAs and Whites were 12.27% (95% CI, 10.58–14.19%) and 7.24% (95% CI, 6.48–8.12%), respectively. This too is consistent with most of the published literature [3]. There are several reasons why AAs have a high diabetes prevalence compared to Whites. These include obesity [34], poverty, and lactose intolerance [35].

Similarly, the prevalence of VDD is also much higher in AAs than in Whites. Our results show that the AA VDD prevalence is 3.4 times higher than the prevalence in Whites. Furthermore, the prevalence of SVDD in AAs is 7-fold higher compared to the SVDD prevalence in Whites. As mentioned previously, the reason why AAs have a higher proportion of VDD is partly attributed to melanin which can act as a natural sunscreen and limit vitamin D production in people with dark skin [9]. Other reasons why AAs have high levels of VDD is thought to be due to obesity [11].

Obesity is a major public health problem in the US, especially among AA women. Fifty-five percent of AAs are clinically obese compared to 38% of White women [36]. This notable disparity in obesity rate is in part the reason why diabetes and VDD prevalence rates are much higher in AAs. Davis postulates that “excessive fat can sequester vitamin D and other fat-soluble vitamins [11].” Wortman et al. [37] gave equal doses of vitamin D supplementation and equal exposure to ultraviolet B irradiation phototherapy to obese women and women with normal weights. The researchers found that the incremental increase in vitamin D was 57% lower in obese women compared to women with normal weights, indicating the association between VDD and obesity.

Our secondary objective is to examine the 25-hydroxyvitamin D test measurements among AAs and Whites and assess the appropriateness of utilizing the same cut-off point for both AAs and Whites in identifying individuals as vitamin D deficient. As evidenced in the “Results” section, AAs exhibited markedly lower 25-hydroxyvitamin D concentrations compared to Whites across the general population, as well as within vitamin D–sufficient and vitamin D–deficient subgroups. For instance, in the general US population, the mean 25-hydroxyvitamin level for AAs was 45.3 (95% CI, 43.5–47.2), while for Whites it was 73.0 (95% CI, 71.1–75.0). It is important to note that despite the significant disparity in the distribution of 25-hydroxyvitamin D measurements between AAs and Whites, the cut-off point (< 50 nmol/L) for diagnosing vitamin D deficiency in clinical practice remains the same for both groups, which may not be justifiable. Furthermore, a reason for the “vitamin D paradox in blacks,” we discussed previously [8], may be that the 25-hydroxyvitamin D test or its associated cut-off point for defining VDD may not be accurate for AAs. Thus, it may be prudent to consider race-specific cut-off points for VDD, taking into account the unique characteristics of each group. Such an approach may help ensure that individuals are accurately categorized based on their risk of VDD and VDD-related conditions relative to the norm for their population.

Powe et al. [38] also raise concerns about the reliability of the 25-hydroxyvitamin D test for AAs. They claim that AAs have low levels of total 25-hydroxyvitamin D and vitamin D–binding protein compared to Whites. However, both AAs and Whites have similar concentrations of estimated bioavailable 25-hydroxyvitamin D, indicating that the 25-hydroxyvitamin D test is a good proxy measure of bioavailability for Whites but may not be an accurate VDD test for AAs. If indeed this holds true, and considering our findings that reveal a notable difference in the distribution of 25-hydroxyvitamin D measurements between AAs and Whites, yet utilizing the same cut-off point to diagnose VDD, this discrepancy may explain why we did not observe any association between VDD and diabetes in AAs.

This study has several strengths. It is based on NHANES which is a well-known, well-designed, and high-quality complex sample survey. Secondly, to our knowledge, this is the first application of a propensity score modeling approach to investigate the association between VDD and diabetes in AAs and Whites in the US using data from a complex sample survey with a large sample size and a rich set of covariates. In our study, we used 31 covariates to develop our propensity score models. Many studies used multivariate regression models to investigate the association between VDD and diabetes. However, such modeling exercises may result in biased VDD-diabetes effect estimates and do not alert researchers when there is no, or poor covariate overlap and this in turn can result in biased inference [39]. In our study, for both the PSM and IPTW algorithms, we obtained good covariate balance (SMD < 0.10) between VDD and vitamin D–sufficient groups and this allows us to obtain a more accurate VDD effect estimate of diabetes [39].

This study has some limitations. As McCarthy et al. [32] mentioned, VDD may be a surrogate measure for poor health or broader socio-economic, dietary, and behavioral variables that influence vitamin D intake. However, in this study, we have adjusted for a rich set of 31 variables, including income, education, body mass index, general health condition, physical activity, and mental health status. Of the 31 variables we used in our complete case data analysis, 20 had less than 1% missing data. The remaining 11 variables had missing data ranging from 4.8 to 12.4%.

Like many statistical techniques used for controlling confounders in studies like ours, the PSM and IPTW algorithms we applied to analyze our dataset can only balance and adjust for observed covariates. Propensity score analysis can result in biased inference when important covariates influencing selection bias are not included in the development of the propensity score model. Unlike randomized clinical trials, propensity score analysis does not adjust for unmeasured covariates, and this can result in biased parameter estimates. However, despite these limitations, our results are consistent with most of the published literature.

In conclusion, our analyses showed that the prevalence of VDD and diabetes were much higher for AAs compared to Whites. And yet, VDD was strongly associated with increased diabetes risk for Whites but not for AAs. We are not exactly sure why VDD is associated with diabetes in Whites but not in AAs. More research is needed to explain why this is the case. However, our data demonstrate that the distribution of 25-hydroxyvitamin D measurements for AAs and Whites for the general population, the vitamin D–sufficient populations, and the VDD population are significantly different. Thus, a possible reason why VDD is not associated with diabetes in AAs may be because the 25-hydroxyvitamin D test may not be an accurate test or the cut-off point for declaring someone as VDD may not be an accurate cut-off point for AAs.

## Electronic supplementary material

Below is the link to the electronic supplementary material.

Supplementary file1 (DOCX 236 KB)

## Data Availability

This study is based on publicly available data, NHANES.

## Appendix

**Table:**

Variable name	Description
Demographic	
Gender	Participant gender (male and female)
Age in years	Age in years of the participant at the time of screening. Individuals 80 and over are top coded at 80 years of age
Race	Recode of reported race and Hispanic origin information. This study only focused on non-Hispanic white and non-Hispanic Black
Marital status	Marital status. Refused and don’t know was recoded to missing
BMI	Body mass index (kg/m**2) = weight / ((height / 100)**2)
Citizenship status	{Are you/Is SP} a citizen of the United States? [Information about citizenship is being collected by the US Public Health Service to perform health related research. Providing this information is voluntary and is collected under the authority of the Public Health Service Act. There will be no effect on pending immigration or citizenship petitions.] Refused and don’t know was recoded to missing
Education	What is the highest grade or level of school {you have/SP has} completed or the highest degree {you have/s/he has} received? Refused and don’t know was recoded to missing
Annual family income	Total family income (reported as a range value in dollars) Refused and don’t know was recoded to missing
Medical	
Ever told you had high blood pressure	Have you ever been told by a doctor or other health professional that you had hypertension, also called high blood pressure? Refused and don’t know was recoded to missing
Doctor told you-high cholesterol level	Have you ever been told by a doctor or other health professional that your blood cholesterol level was high? Refused and don’t know was recoded to missing
Diastolic: blood pressure	Diastolic: blood pressure (mm Hg) the third reading
Systolic: blood pressure	Systolic: blood pressure (mm Hg) the third reading
General health condition	General health condition. Refused and don’t know was recoded to missing
Total cholesterol	Total cholesterol (mg/dL)
Ever told you had cancer/malignancy	Have you ever been told by a doctor or other health professional that you had cancer or a malignancy of any kind? Refused and don’t know was recoded to missing
Ever told had congestive heart failure	Has a doctor or other health professional ever told you that you had congestive heart failure? Refused and don’t know was recoded to missing
Ever told you had coronary heart dx	Has a doctor or other health professional ever told you that you had coronary heart disease? Refused and don’t know was recoded to missing
Ever told you had angina	Has a doctor or other health professional ever told you that you had angina/angina pectoris? Refused and don’t know was recoded to missing
Ever told you had heart attack	Has a doctor or other health professional ever told you that you had a heart attack (also called myocardial infarction? Refused and don’t know was recoded to missing
Ever told you had a stroke	Has a doctor or other health professional ever told {you/SP} that you had a stroke? Refused and don’t know was recoded to missing
Ever told you had emphysema	Has a doctor or other health professional ever told {you/SP} that you had emphysema? Refused and don’t know was recoded to missing
Behavioral	
Have little interest in doing things	Over the last 2 weeks, how often have you been bothered by the following problems: little interest or pleasure in doing things? Refused and don’t know was recoded to missing
Feeling down, depressed, or helpless	Over the last 2 weeks, how often have you been bothered by the following problems: feeling down, depressed, or hopeless? Refused and don’t know was recoded to missing
Trouble sleeping or sleeping too much	Over the last 2 weeks, how often have you been bothered by the following problems: trouble falling or staying asleep, or sleeping too much? Refused and don’t know was recoded to missing
Feeling bad about yourself	Over the last 2 weeks, how often have you been bothered by the following problems: feeling bad about yourself—or that you are a failure or have let yourself or your family down? Refused and don’t know was recoded to missing
Trouble concentrating on things	Over the last 2 weeks, how often have you been bothered by the following problems: trouble concentrating on things, such as reading the newspaper or watching TV? Refused and don’t know was recoded to missing
Thought you would be better off dead	Over the last 2 weeks, how often have you been bothered by the following problems: Thoughts that you would be better off dead or of hurting yourself in some way? Refused and don’t know was recoded to missing
Vigorous work activity	Does {your/SP’s} work involve vigorous-intensity activity that causes large increases in breathing or heart rate like carrying or lifting heavy loads, digging or construction work for at least 10 min continuously? Refused and don’t know was recoded to missing
Moderate work activity	Does {your/SP’s} work involve moderate-intensity activity that causes small increases in breathing or heart rate such as brisk walking or carrying light loads for at least 10 min continuously? Refused and don’t know was recoded to missing
Walk or bicycle	{Do you/Does SP} walk or use a bicycle for at least 10 min continuously to get to and from places? Refused and don’t know was recoded to missing
Smoked at least 100 cigarettes in life	{Have you/Has SP} smoked at least 100 cigarettes in {your/his/her} entire life? Refused and don’t know was recoded to missing

## Code Availability

Code can be available upon request.
