# Supplementary material for: The Association Between Vitamin D Deficiency and Diabetes in Adult African Americans and Whites: An NHANES Study
Source: J Racial Ethn Health Disparities. 2024 Sep 23;12(5):3401–16. doi: 10.1007/s40615-024-02144-4 (PMC12446391; doi:10.1007/s40615-024-02144-4)
Supplement: Supplementary file 1 — Supplementary file1 (DOCX 236 KB) [file 40615_2024_2144_MOESM1_ESM.docx]

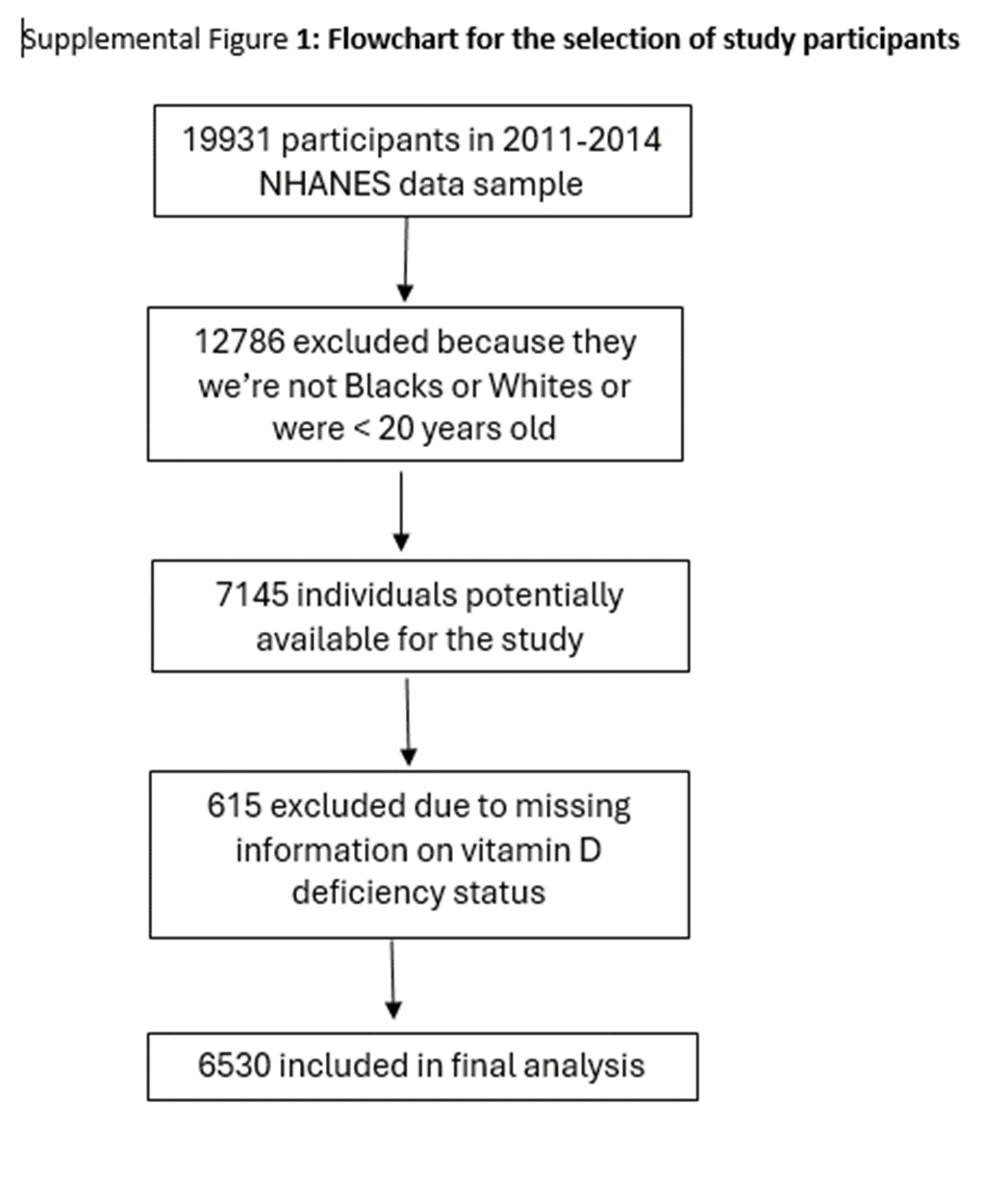


Supplemental Figure 2: Standardized Mean Differences for the IPTW Model^1^ riable names see Table 1.


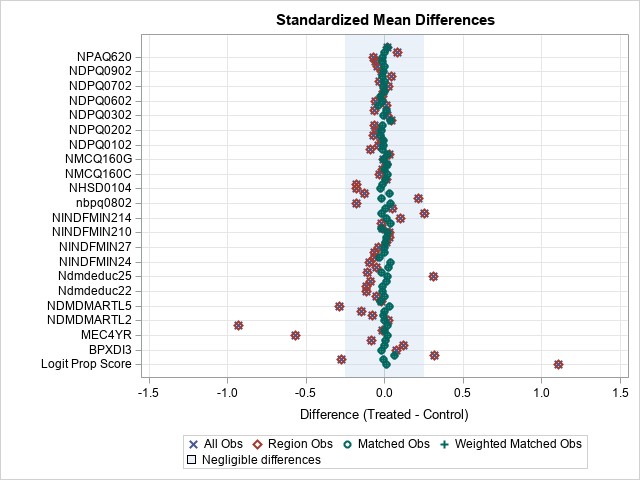


^1^ For the definition of variable names, see Table 1.

**Supplemental Figure 3: Standardized Mean Differences for the IPTW Model^1^** -for variable names see Table 1.

354
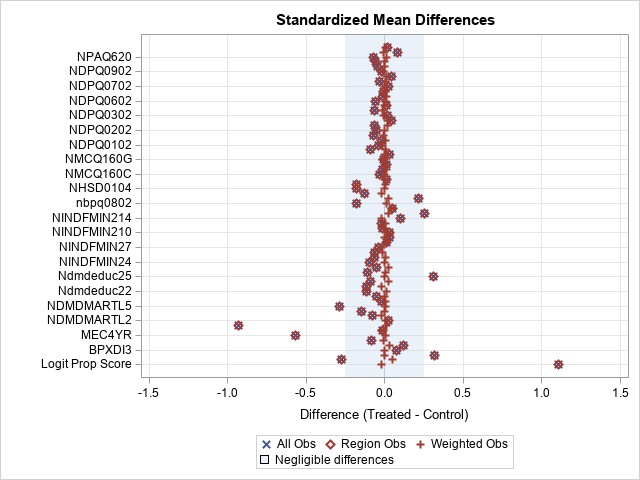


355 ^1^ For a definition of variable names, see Table 1.
